# A Mucin-5B variant associated with idiopathic pulmonary fibrosis may prolong survival in the general population

**DOI:** 10.1172/JCI211620

**Published:** 2026-11-16

**Authors:** Mitchell J. Riley, Jacob E. Simmering, Michael J. Welsh

**Affiliations:** 1 Department of Internal Medicine,; 2 Department of Molecular Physiology and Biophysics, Pappajohn Biomedical Institute, Roy J. and Lucille A. Carver College of Medicine, University of Iowa, Iowa City, Iowa, USA.

**Keywords:** Genetics, Public Health, Pulmonology, Genetic diseases

**To the Editor:** Idiopathic pulmonary fibrosis (IPF) is a rare fibrotic, interstitial lung disease that progressively destroys the lung. Genome-wide association studies identified a common single-nucleotide polymorphism in the *MUC5B* gene promoter (rs35705950, *T* allele) in ≥ 50% of people with IPF (1). The *MUC5B* *G* allele is the common allele; the *T* allele occurs in approximately 20% of the general population and is associated with an approximately 5-fold increased risk of developing IPF. Throughout, the *T* allele refers to both *GT* and *TT* genotypes. The high prevalence and low penetrance of a variant conferring an elevated risk for a lethal disease raised the question of whether the *T* allele might provide an evolutionary benefit. Two observations brought further suspicion about this possibility: IPF patients carrying the *T* allele are reported to have a later onset of disease and longer survival after diagnosis (2). Therefore, we tested the hypothesis that the *T* allele is associated with a survival advantage.

Previous studies focused on people who have established IPF. To evaluate the general population for the risk associated with the *T* allele, we analyzed data from 232,401 participants in the *All of Us* Research Program’s database (Supplemental Table 1; supplemental material available online with this article; https://doi.org/10.1172/JCI211620DS1). The *T* allele was associated with a four-fold increased hazard ratio (HR) of developing IPF (Figure 1A and Supplemental Table 2), consistent with previous reports (1).

Because smoking and male sex have been associated with an increased risk of IPF, we asked if they confound assessment of *T* allele effects. Smoking was associated with an increased IPF HR, and male sex had a time-varying HR for IPF, suggesting that the relative IPF hazard between males and females varied by age (Supplemental Table 2). However, adjustment for smoking, sex, and predicted ancestry did not change the hazard associated with the *T* allele (Figure 1A).

We found that participants with the *T* allele were diagnosed at a younger age than participants with *GG* alleles (Figure 1B). This contrasts with a previous study of a group of patients with IPF, which reported that patients with *T* allele were older at diagnosis (2). By studying people enrolled before an IPF diagnosis, our analysis avoids biases associated with competing risks (Supplemental Material).

To test if the *T* allele is associated with a survival advantage in the general population, we assessed all-cause mortality. The *T* allele was associated with decreased all-cause mortality (Figure 1C and Supplemental Table 3). The associated lower hazard of death in people with the *T* allele persisted after testing for potential confounding relationships with smoking, sex, and predicted ancestry. Both smoking and male sex had time-varying HR (Supplemental Table 3). Kaplan-Meier analysis revealed that participants with a *T* allele had ≥ 1% increase in survival between ages 55–78 and ≥ 2% between ages 79–95 (Figure 1D). A UK Biobank study serendipitously also found the *T* allele associated with improved male survival from all-cause mortality (3). These results reveal a potential association between the *MUC5B* *T* allele and survival. Validation of these results will require studies of additional databases.

Might the *T* allele confer an evolutionary benefit even though its association with increased survival appears late in life? First, theory and evidence suggest that prolonged postreproductive lifespans can provide fitness advantages in humans and selected other species (4). Second, the average enrollment age in the *All of Us* database was 53 years, preventing assessment in children and young adults. Thus, we speculate that the relatively high frequency of the *T* allele reflects survival advantages in late and/or early life.

What might account for a survival benefit? For most of history, infectious diseases have been the leading cause of mortality. In respiratory airways, MUC5B contributes to mucociliary transport, a key defense against inhaled pathogens. The *T* allele increases MUC5B expression (1), raising the possibility that elevated MUC5B secretion enhances airway host defense. Accordingly, UK Biobank and Million Veterans Program studies reported an association between the *T* allele and fewer COVID-19–related hospitalizations (5, 6). We found that the *T* allele was not associated with a reduced risk of COVID-19 but was associated with a reduced hospitalization risk (Supplemental Figure 1). We found no difference in risk for the combined group of all lung infections or accompanied hospitalizations. We speculate that the *T* allele may confer protection against some severe respiratory infections. Additional studies may reveal further insights into these interesting results.

Our study has limitations. (a) Numbers of death records are small and lack cause-of-death data. (b) Relatively few participants had IPF, and diagnoses were determined from participant electronic health records. (c) The study population was limited to participants of European or Admixed-American ancestry (Supplemental Material) because *All of Us* data dissemination policy prohibits reporting case numbers below 20, which restricted our analysis to these ancestries. (d) The database only includes participants ≥ 18 years old; thus, we cannot exclude the possibility that the *T* allele–associated advantage requires surviving to that age.

The results suggest an evolutionary trade-off, with the IPF risk outweighed by prolonged survival. To illustrate, consider 10,000 people with *GG* alleles and 10,000 people with a *T* allele. Compared with GG alleles, the T allele population would be expected to have approximately 10 more people with IPF (the difference in incidence for the T allele versus the GG alleles is ~9.8:10,000, Supplemental Table 1). Conversely, the T allele population would be expected to have approximately 200 more people survive to age 79 (the average USA life expectancy) (2% of 10,000, Figure 1D). Thus, at a population level, the *T* allele may confer greater evolutionary benefit than risk.

## Manuscript information

### Conflict of interest.

The authors have declared that no conflict of interest exists.

### Funding.

This study was supported by the following:

- The Howard Hughes Medical Institute.

## Supplementary Material

Supplemental data

Supporting data values

## Figures and Tables

**Figure 1:**
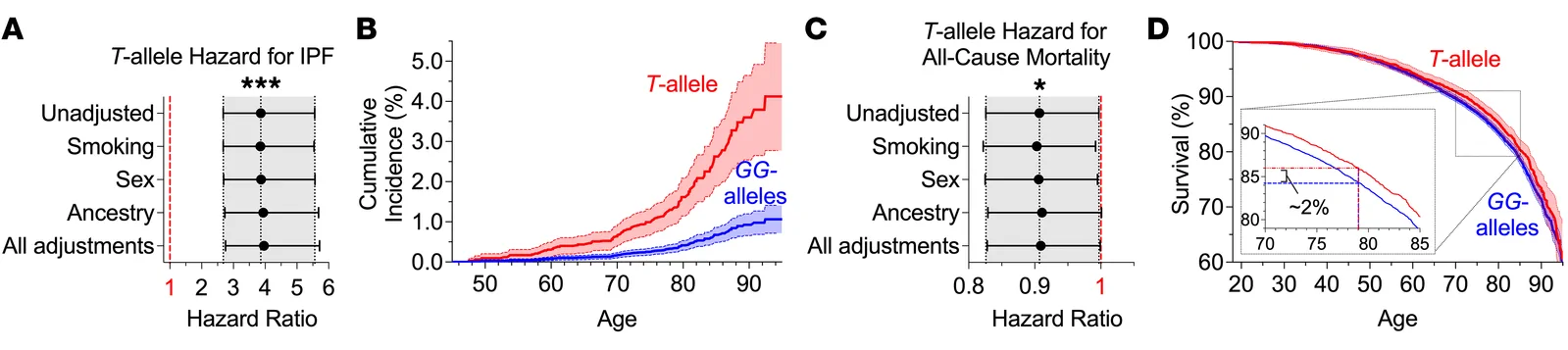
Assessment of IPF diagnosis and all-cause mortality risk. Throughout, red indicates participants with a *T* allele, blue with *GG* alleles, whiskers indicate 95% confidence intervals, shaded regions indicate 95% confidence bands, and **A**–**C** used a Wald statistical test. (**A**) *T* allele HR for IPF diagnosis unadjusted and adjusted for the indicated covariates (4.0, [95% CI, 2.7–5.7]). ***, *P* < 0.001 for the all adjustments estimate. (**B**) Fine-Gray competing risks cumulative incidence curve, HR = 3.9, *P* < 0.001. (**C**) *T* allele HR for all-cause mortality, unadjusted and adjusted for the indicated covariates (0.91, [0.83–0.999]); * *P* = 0.048 for the all adjustments estimate). (**D**) Kaplan-Meier survival analysis. *P* = 0.043 by log-rank test. Inset shows increased survival associated with the *T* allele at age 79.

## Version 1 11/16/2026

Electronic publication

## References

[1] Seibold MA (2011). A common MUC5B promoter polymorphism and pulmonary fibrosis. N Engl J Med.

[2] van der Vis JJ (2023). MUC5B rs35705950 minor allele associates with older age and better survival in idiopathic pulmonary fibrosis. Respirology.

[3] Park J (2025). Rare genetic associations with human lifespan in UK Biobank are enriched for oncogenic genes. Nat Commun.

[4] Croft DP (2015). The evolution of prolonged life after reproduction. Trends Ecol Evol.

[5] van Moorsel CHM (2021). The MUC5B promoter polymorphism associates with severe COVID-19 in the European population. Front Med (Lausanne).

[6] Verma A (2022). A MUC5B gene polymorphism, rs35705950-T, confers protective effects against COVID-19 hospitalization but not severe disease or mortality. Am J Respir Crit Care Med.

