# Supplementary material for: A Mucin-5B variant associated with idiopathic pulmonary fibrosis may prolong survival in the general population
Source: J Clin Invest. 2026 Nov 16;136(22):e211620. doi: 10.1172/JCI211620 (PMC13767610; doi:10.1172/JCI211620)
Supplement: Supplementary file 1 [file jci-136-211620-s255.pdf]

SUPPLEMENTAL MATERIAL

A Mucin-5B variant associated with idiopathic pulmonary fibrosis may prolong survival in the general population

Mitchell J. Riley[1], Jacob E. Simmering[1], Michael J. Welsh[1,2]

[1]Department of Internal Medicine, Pappajohn Biomedical Institute,
University of Iowa, Iowa City, IA, USA. [2]Department of Molecular Physiology and Biophysics,
Roy J. and Lucille A. Carver College of Medicine, University of Iowa, Iowa City, IA, USA

Communications:
Michael J. Welsh
169 Newton Rd, 6332 PBDB
Iowa City, IA 52242
Phone 319-335-7619
michael-welsh@uiowa.edu

Contents:

Supplemental Tables 1-3

Supplemental Figure

Methods

References

SUPPLEMENTAL TABLES

	All (N)	*GG*-alleles, N (%)	*T*-allele, N (%)
**Participants**	232,401	188,239 (81%)	44,162 (19%)
**IPF diagnoses**	116	59 (51%)	57 (49%)
**IPF incidence**	5:10,000	3.1:10,000	12.9:10,000
**Death records**	2,929	2,396 (82%)	533 (18%)

Table S1. Number of participants, IPF diagnoses, IPF incidence, and death records across *MUC5B* genotypes.

Hazard ratios (IPF diagnosis)	Hazard ratio	95% CI	P value
**T allele**	3.963	2.750 – 5.712	<0.001
**Smoking**	1.825	1.197 – 2.782	0.005
**Male sex***	0.937	0.520 – 1.690	0.829
**Admixed-American ancestry**	1.632	0.955 – 2.788	0.073

Table S2. Hazard ratios for IPF risk. IPF diagnosis hazard ratios for *T*-allele, smoking, male sex at age 65, and Admixed-American ancestry. *The time coefficient for male sex was (1.103, [1.048-1.161], $p < 0.001$), corresponding to the hazard ratio $0.937\times(1.103)^{(\text{age} - 65)}$.

Hazard ratios (All-cause mortality)	Hazard ratio	95% CI	P value
**T allele**	0.909	0.828 – 0.999	0.048
**Smoking***	1.917	1.741 – 2.112	<0.001
**Male sex†**	1.535	1.424 – 1.656	<0.001
**Admixed-American ancestry**	1.138	1.028 – 1.260	0.012

Table S3. Hazard ratios for all-cause mortality. All-cause mortality hazard ratios for *T*-allele, smoking at age 65, male sex at age 65, and Admixed-American ancestry. *The time coefficient for smoking was (0.990, [0.983-0.997], $p = 0.006$), corresponding to the hazard ratio $1.917\times(0.990)^{(\text{age} - 65)}$. †The time coefficient for male sex was (0.990, [0.985-0.995], $p < 0.001$), corresponding to the hazard ratio $1.535\times(0.990)^{(\text{age} - 65)}$.

SUPPLEMENTAL FIGURE

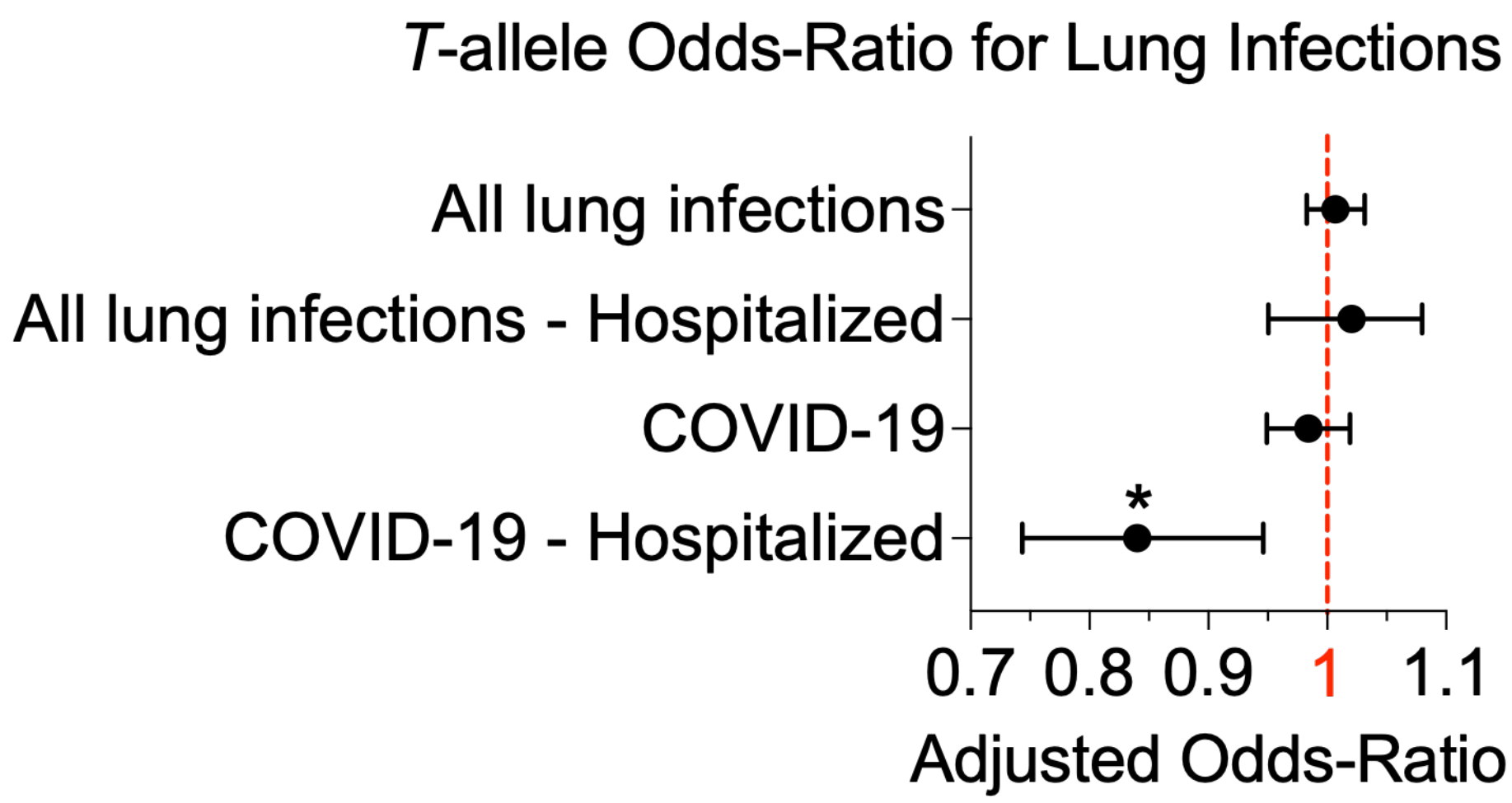

Figure S1. Risk of any lung infection and COVID-19. Data are adjusted odds-ratios with whiskers indicating 95% percent confidence intervals. The fraction of participants with *GG* alleles and any lung infection was 46,909/187,582 and the fraction of those hospitalized was 6,482/187,582. The fraction of participants with the *T*-allele and any lung infection was 11,274/44,013 and the fraction of those hospitalized was 1,552/44,013. The fraction of participants with *GG* alleles and COVID-19 infection was 19,019/187,582 and the fraction of those hospitalized was 1,721/187,582. The fraction of participants with the *T*-allele and COVID-19 infection was 4,375/44,013 and the fraction of those hospitalized was 320/44,013. *, indicates p<0.01. ICD-9 and ICD-10 codes used for any lung infection were: 466, 466.0, 466.1, 466.11, 466.19, 480, 480.0, 480.1, 480.2, 480.3, 480.8, 480.9, 481, 482, 482.0, 482.1, 482.2, 482.3, 482.30, 482.31, 482.32, 482.39, 482.4, 482.40, 482.41, 482.42, 482.49, 482.8, 482.81, 482.82, 482.83, 482.84, 482.89, 482.9, 483, 483.0, 483.1, 483.8, 484, 484.1, 484.3, 484.5, 484.6, 484.7, 484.8, 485, 486, 487, 487.0, 487.1, 488.0, 488.01, 488.02, 488.1, 488.11, 488.12, 488.81, 488.82, 510, 510.0, 510.9, 513.0, A48.1, J09.X, J09.X1, J09.X2, J10, J10.00, J10.01, J10.08, J10.1, J11, J11.00, J11.08, J11.1, J12, J12.0, J12.1, J12.2, J12.3, J12.81, J12.82, J12.89, J12.9, J13, J14, J15, J15.0, J15.1, J15.20, J15.21, J15.211, J15.212, J15.29, J15.3, J15.4, J15.5, J15.6, J15.7, J15.8, J15.9, J16.0, J16.8, J17, J18, J18.0, J18.1, J18.2, J18.8, J18.9, J20, J20.0, J20.1, J20.2, J20.3, J20.4, J20.5, J20.6, J20.7, J20.8, J20.9, J21, J21.0, J21.1, J21.8, J21.9, J22, J85.0, J85.1, J85.2, J86, J86.0, J86.9, U07.1. ICD-10 codes used for COVID-19 were: J12.82 and U07.1.

SUPPLEMENTAL METHODS

All of Us Research Program. Started in 2017, the program enrolls voluntary participants who live in the United States and are at least 18 years of age.

Study population. The number of participants in the *All of Us* database with whole genome sequencing and electronic health records is 317,964. Participants must be living to enroll in *All of Us*, and since patients with IPF and the *T*-allele live longer than IPF patients without the *T*-allele, we restrict IPF diagnoses to 180 days after *All of Us* enrollment to avoid biasing our IPF cohort toward participants with the *T*-allele or *GG*-alleles. Participants with incomplete smoking or sex-assigned-at-birth survey data were excluded, as analyses require complete data for these variables, leaving 305,838 participants. Next, we stratified the remaining participants by predominant ancestry, which includes European, African, Admixed-American, South Asian, East Asian, and Middle Eastern. Participants with Middle Eastern and South Asian ancestry had sample sizes of fewer than 1,200 and 4,000, respectively, and no diagnoses of IPF; therefore, they were excluded from the main analyses. The percentage of people with at least one *MUC5B* *T*-allele was only 4.2% for African ancestry and 1.8% for East Asian ancestries, consistent with percentages derived from gnomAD and earlier studies (1, 2), and thus were also excluded from the main analyses. We excluded them because our goal was to assess the contribution of *MUC5B* genotype to the risk of IPF and all-cause mortality. Moreover, *All of Us* restricts reporting participants counts fewer than 20, stating "No participant count of 1 to 20 can be published or distributed directly (a count of 0 is permitted); and no data or statistics can be reported that allow a participant count of 1 to 20 to be derived from other reported cells or information, including in text, tables, or figures. This includes the use of percentages or other mathematical formulas that in combination would allow an individual to deduce a participant count of less than 20" (3). Due to this, we avoided performing analysis that would require reporting participant counts fewer than 20 or allow for back-calculation to a participant count fewer than 20. Thus, the cohort used for analyses consisted of participants with predominantly European and Admixed-American ancestry, among whom 21.1% and 12.6%, respectively, carried at least one *T*-allele, resulting in a total cohort size of 232,401 participants (Table S1). Participants classified as Admixed-American have a highly heterogeneous, admixed ancestry, and ancestry inference resolves these individuals' admixed ancestry into Amerindigenous, African, and European haplotypes (4). European and Admixed-American ancestries are lumped together to avoid reporting IPF diagnoses fewer than 20 for participants of Admixed-American ancestry.

Risk and incidence of IPF diagnosis. IPF diagnoses were identified by Systematized Nomenclature of Medicine - Clinical Terms (SNOMED CT) concept 700250006 (5). Each participant was defined to be at risk until one of the following outcomes: diagnosis of IPF, death, or October 1, 2023, the last data collection date for *All of Us* Controlled Tier v8 (6). Covariates were *MUC5B* *T*-allele, smoking status (≥20 pack-years), sex-assigned-at-birth, and predicted ancestry. Smoking and sex were chosen as covariates because previous studies associated smoking and male sex to a higher risk of IPF diagnosis (7). Predicted ancestry was used to adjust for differing allele frequencies of the *MUC5B* *T*-allele across populations and socio-economic status. Smoking history and sex were determined from surveys administered by the NIH at participant enrollment and are therefore subject to limitations inherent to self-reported data, including potential recall and reporting bias. Predicted ancestry was determined by genetic

similarity to the reference populations Human Genome Diversity Project and 1000 Genomes Project, with more details provided in (8).

Survival and risk of all-cause mortality
Time at risk for death began at participant enrollment. Death confirmation was provided with a date of death by *All of Us*, but cause of death was not given. Each participant was considered at risk of death until his or her death date or October 1, 2023. Covariates used were *MUC5B T*-allele, smoking status (≥20 pack-years), sex-assigned-at-birth, and predicted ancestry. Smoking and sex were chosen because previous studies reported that smokers and men had a higher risk of all-cause mortality (9, 10). Predicted ancestry was chosen as a covariate for the same reason as above.

Risk of lung infection and COVID-19 infection
To determine risk of lung infection or severe lung infection, we computed adjusted odds-ratios for all lung infections, COVID-19 infections, and hospitalizations from all lung or COVID-19 infections (Figure S1). ICD-9 and ICD-10 codes were used to determine history of infection. Those codes linked to a visit description of "Inpatient Visit," "Inpatient Hospital," "Emergencry Room and Inpatient Visit," or "Intensive Care" were used for hospitalization events. Participants who died prior to 2021 were removed from these analyses to avoid a protective bias from participants that were never at risk for COVID-19 infection. Covariates used were *MUC5B T*-allele, age, smoking status (≥20 pack-years), sex-assigned-at-birth, and predicted ancestry. Age was calculated as from a particpant's birth year up to his or her death year or October 1, 2023.

Sex as a biological variable. The study population consisted of 118,302 males and 187,536 females. Sex was included as a covariate in our analyses. Males have been reported to be at higher risk for IPF and all-cause mortality.

Statistics. A Fine-Gray model was used to compute the cumulative incidence of IPF diagnosis between people with or without the *T*-allele while controlling for the competing risk of death. All-cause mortality survival probabilities in people with or without the *MUC5B T*-allele were computed using R's *survival* package with the Surv function. Adjusted and unadjusted hazard ratios for both IPF diagnosis and all-cause mortality events were computed with a Cox proportional hazards model using R's *survival* package. Main effects included were *T*-allele, sex, smoking status, and predicted ancestry. For IPF hazard, the sex covariate violated the proportional hazards assumption (Schoenfeld residuals, $p = 4.2 \times 10^{-4}$, global $p = 0.0031$) and was therefore modeled with a time-varying coefficient, arbitrarily centered around age 65 (Table S2). Similarly, for all-cause mortality hazard, sex and smoking violated the proportional hazards assumption (Schoenfeld residuals: sex $p = 3.8 \times 10^{-5}$, smoking $p = 0.0015$, global $p = 4.9 \times 10^{-5}$) and were therefore both modeled with time-varying coefficients, both arbitrarily centered around age 65 (Table S3). We report the unadjusted hazard conferred by the *T*-allele in Figures 1A, 1C as well as its adjusted hazard including each individual covariate and all covariates. R's glm function was used to compute adjusted odds-ratios for lung infection risk (Figure S1). For future studies, we also conducted a power analysis using Schoenfeld's formula (11). The number of death events required to detect an HR of 0.91 at the 0.05 significance level with 80% power and 19% exposure prevalence is 5,727.

Study approval. All participant data was acquired by the NIH, whose policy on gathering human subject data is available on researchallofus.org. Participant data are de-identified and only allowed to be accessed via the *All of Us* Researcher Workbench to ensure participant privacy and adequate data security. Participation in *All of Us* is voluntary. Access to *All of Us* was acquired via a non-material transfer agreement between the University of Iowa's Division of Sponsored Programs and the NIH's *All of Us* Research Program.

Data availability. All code is stored in the *All of Us* Researcher Workbench. Access to the *All of Us* Researcher Workbench requires special permission. More information can be found at allofus.nih.gov. We provide supporting values for the figures in a Microsoft Excel spreadsheet.

Author contributions. Designed studies and interpreted data: MJR, JES, MJW. Performed analysis: MJR. Wrote the manuscript: MJR and MJW. All authors approved the final version of the manuscript.

AI disclosure. AI assisted in identifying ICD codes for lung infections. The AI tool used was Claude Version 1.46388.1from August 24, 2026 to September 3, 2026.

ACKNOWLEDGEMENTS
We gratefully acknowledge *All of Us* participants for their contributions, without whom this research would not have been possible. We thank the National Institutes of Health's *All of Us* Research Program for making available the participant data examined in this study. We thank Dr. David A. Schwartz for helpful comments.

SUPPLEMENTAL REFERENCES

1. Genome Aggregation Database (gnomAD) v4.1. Broad Institute. https://gnomad.broadinstitute.org/. Updated October 3, 2025. Accessed April 20, 2026.
2. Peljto AL, et al. Idiopathic pulmonary fibrosis risk loci in East Asian populations mirror those of European populations. *Am J Respir Crit Care Med*. 2026;212(7):1522-1532.
3. All of Us Research Program User Support. Data and Statistics Dissemination Policy. https://support.researchallofus.org/hc/en-us/articles/22346276580372-Data-and-Statistics-Dissemination-Policy. Updated January 18, 2024. Accessed July 22, 2026.
4. Wilson M, Tiao G, Atkinson E. Local ancestry inference for Latino/Admixed American samples in gnomAD. gnomAD browser website. https://gnomad.broadinstitute.org/news/2021-12-local-ancestry-inference-for-latino-admixed-american-samples-in-gnomad/. Published December 1, 2021. Accessed July 14, 2026.
5. Overview of SNOMED CT. National Library of Medicine website. https://www.nlm.nih.gov/healthit/snomedct/snomed_overview.html. Accessed May 13, 2026.
6. National Institutes of Health. Curated Data Repository (CDR) version 8 release notes. All of Us Research Program User Support website. https://support.researchallofus.org/hc/en-us/articles/30294451486356-Curated-Data-Repository-CDR-version-8-Release-Notes. Published February 4, 2025.
7. Podolanczuk AJ, et al. Idiopathic pulmonary fibrosis: state of the art for 2023. *Eur Respir J*. 2023;61(4):2200957.
8. All of Us Research Program. Genomic QC report CDRv8 [PDF]. All of Us Research Program User Support website. https://support.researchallofus.org/hc/en-us/article_attachments/40491583732116. Published 2025.
9. Doll R, et al. Mortality in relation to smoking: 50 years' observations on male British doctors. *BMJ*. 2004;328(7455):1519.
10. Kalben BB. Why men die younger: causes of mortality differences by sex. *N Am Actuar J*. 2000;4(4):83-111.
11. Schoenfeld DA. Sample-size formula for the proportional-hazards regression model. *Biometrics*. 1983;39(2):499–503.
